# Range-wide genetic structure and demographic history in the bat ectoparasite *Cimex adjunctus*

**DOI:** 10.1186/s12862-016-0839-1

**Published:** 2016-12-07

**Authors:** Benoit Talbot, Maarten J. Vonhof, Hugh G. Broders, Brock Fenton, Nusha Keyghobadi

**Affiliations:** 1 Department of Biology, University of Western Ontario, 1151 Richmond Street, London, ON Canada; 2 Department of Biological Sciences, Western Michigan University, 1903 W Michigan Avenue, Kalamazoo, MI USA; 3 Department of Biology, Saint Mary’s University, 923 Robie Street, Halifax, NS Canada

**Keywords:** AMOVA, Approximate Bayesian computation, Bayesian skyline plot, *CO1*, Genetic clustering, Isolation by distance, Phylogeography

## Abstract

**Background:**

Evolutionary histories of parasite and host populations are intimately linked such that their spatial genetic structures may be correlated. While these processes have been relatively well studied in specialist parasites and their hosts, less is known about the ecological and evolutionary consequences of relationships between generalist ectoparasites and their hosts. The aim of this study was to investigate the genetic structure and demographic history of a bat ectoparasite, *Cimex adjunctus*, whose host affinity is weak but the biology of the potential hosts have been well studied. This ectoparasite has been hypothesized to rely on its hosts for dispersal due to its low inherent dispersal potential. Here we describe genetic diversity and demographic history in *C. adjunctus* through most of its range in North America. We investigated variation at the cytochrome c oxidase 1 mitochondrial gene and nine microsatellite markers, and tested the prediction that genetic diversity in *C. adjunctus* is spatially structured. We also tested the prediction that demographic history in *C. adjunctus* is characterized by range and demographic expansion as a consequence of post-Pleistocene climate warming.

**Results:**

We found stronger spatial structuring of genetic diversity in *C. adjunctus* than has been quantified in two of its hosts, but contrast in amount of variation explained by host association with different genetic markers (i.e., nuclear vs mitochondrial DNA). Also, *C. adjunctus’* history is not primarily characterized by demographic and range expansion, as is the case with two of its key hosts.

**Conclusions:**

Our study shows different patterns of genetic structure and demographic history in *C. adjunctus* than have been detected in two of its key hosts. Our results suggest an effect of a loose parasite-host relationship and anti-parasitism strategies on genetic structure and post-Pleistocene recovery of population size.

**Electronic supplementary material:**

The online version of this article (doi:10.1186/s12862-016-0839-1) contains supplementary material, which is available to authorized users.

## Background

Parasites, through effects on host survival and reproduction, can modify the morphology, life history and behavior of their hosts. Parasites may also influence the dynamics of host populations thereby shaping communities [1]. Hosts in turn may also have important effects on their parasites. Many parasite species, whether endoparasites or ectoparasites, remain closely associated with their hosts through much of their life cycle [2], and often rely on their hosts for dispersal. Dispersal, in turn, influences gene flow and therefore genetic structure and diversity of a species; across a broad range of taxa, less dispersal is associated with increased spatial structure and differentiation [3]. Not surprisingly, spatial genetic structure of a parasite frequently reflects dispersal of its host. For example, population genetic structure of parasitic nematodes of cattle, sheep and white-tailed deer is explained by host movements [4]. However, relative to their hosts, parasites often show higher levels of genetic differentiation. As such, analysis of the trematode parasite (*Pagioporus shawi*) permitted more detailed information on population assignments in its host, the steelhead trout (*Oncorhynchus mykiss*) than could be obtained by examining genetic variation in the host itself [5]. In addition to dispersal, parasites and hosts may have experienced correlated demographic and range dynamics [6, 7] which will also be reflected in their population genetic structure; for instance, patterns of genetic variation among populations of the parasitic nematode *Heligmosomoides polygyrus* have revealed demographic and historic events affecting its host, the field mouse *Apodemus sylvaticus* [8]. Furthermore, differences in regional abundance of two *Apodemus* species likely caused differentiation of both the *Apodemus* host and their *Heligmosomoides* parasite species [9].

However, it has recently been shown that a strong link between host dispersal and parasite genetic structure is not ubiquitous, and depends on factors that include the degree of association with the host and host mobility [10]. Here, we investigated spatial genetic structure and past demography of an ectoparasite that is associated with highly mobile flying hosts, and would be considered a weak generalist based on its association with a number of different host species that are closely related to each other [10]. Our study complements a body of work on spatial genetic structure and phylogeography of various ectoparasites associated with hosts having higher mobility [11–13].

Insects in the genus *Cimex* (Order: Hemiptera) are temporary ectoparasites of homeothermic animals. They do not remain on their host at all times but rather remain in nests or roosts between blood meals [14]. Most *Cimex* species are associated exclusively with bats, while a few associate with a more diverse range of hosts [14–16]. *Cimex adjunctus* is a widespread ectoparasite of bats in North America, occurring from the eastern seaboard to the Rocky Mountains, and from Labrador and the Northwest Territories south to Texas [14]. It parasitizes a number of bat species, including the big brown bat (*Eptesicus fuscus*) and the little brown myotis (*Myotis lucifugus*), two species that often roost in buildings [17–19]. The generation time of *C. adjunctus* is unknown, but is likely similar to that of the common bed bug *C. lectularius*, which can range from two to 12 generations a year depending on monthly temperatures [14], and is certainly much shorter than that of its hosts.

Usinger [14] proposed that *Cimex* species have a very low inherent capacity for dispersal over long distances, on the scale of kilometers. He thought it unlikely that adult *Cimex* species disperse on their own. He therefore hypothesized that *Cimex* species can disperse occasionally attached to a host’s body [14]. Previous studies of genetic diversity of the big brown bat and little brown myotis in North America have reported high within-site genetic variation and generally low among-site differentiation, although there are differences between patterns at nuclear and mitochondrial markers (*E. fuscus*, [20, 21]; *M. lucifugus*, [22–25]). Overall, these studies indicate that high levels of gene flow are maintained over long distances in both bat species, while genetic structuring of mitochondrial variation suggests a higher degree of female than male philopatry. For *C. adjunctus*, likely only a fraction of host dispersal events result in successful parasite dispersal so gene flow may be lower in *C. adjunctus* relative to these two host species. Furthermore, *C. adjunctus* may experience frequent extirpation and recolonization events. Bartonička and Růžičková [26] identified bat bug load as a possible cause of roost-switching in bats, with numbers of bats dropping as the population of *C. pipistrelli* reaches a high. They also found the appearance of *C. pipistrelli* 21 to 56 days after the first bat visit in any given roost. Since *C. adjunctus*, like *C. pipistrelli,* does not stay on the host between blood meals, sudden host population decreases within roosts might drive local extirpation events.

Although different ectoparasite races are often associated with different host species [27–29], high gene flow among populations associated with different host species has also been documented. In Europe, *Cimex pipistrelli* is morphologically, but not genetically, differentiated among bat host species [30]. This suggests possible morphological plasticity, but high gene flow, among individuals associated with different host species. In North America, we might also expect gene flow among *C. adjunctus* populations on different host species. Many different North American bat species temporarily roost together for short intervals during the night, such as many *Myotis* species, including *M. lucifugus,* and *E. fuscus* [31], potentially facilitating host switching by *C. adjunctus*.

Much of North America was unsuitable for many bat species during the last Pleistocene glacial maximum, and both *M. lucifugus* and *E. fuscus* are hypothesized to have expanded their ranges from glacial refugia. Dixon [32] suggested that little brown myotis populations currently in Minnesota have dispersed from a single large southeastern US glacial refugium, and Neubaum et al. [21] suggested that big brown bat populations have dispersed from several eastern and western US glacial refugia into what is now Colorado. Range and demographic expansion in little brown myotis has also been proposed on the east coast of Canada [22]. We expect that the potential dependence of *C. adjunctus* on its host species for long-distance dispersal and colonization may have contributed to broadly congruent patterns of historical range expansion over large spatial scales.

We investigated the spatial genetic structure and phylogeography of *C. adjunctus* across its range in North America. Because of its comparatively shorter generation time, the likelihood that only a fraction of bat dispersal events may result in ectoparasite gene flow, and the potential for local extirpations, we predicted stronger spatial genetic structure in *C. adjunctus* relative to its hosts. Because of the potential for movement among host species, we also examined differentiation among populations found on different host species. Finally, based on the hypothesis that post-Pleistocene climate warming had similar effects on the demographic history of *C. adjunctus* as that of its hosts, we predicted genetic signatures of demographic and range expansion in *C. adjunctus*.

## Results

**Fig. 1:**
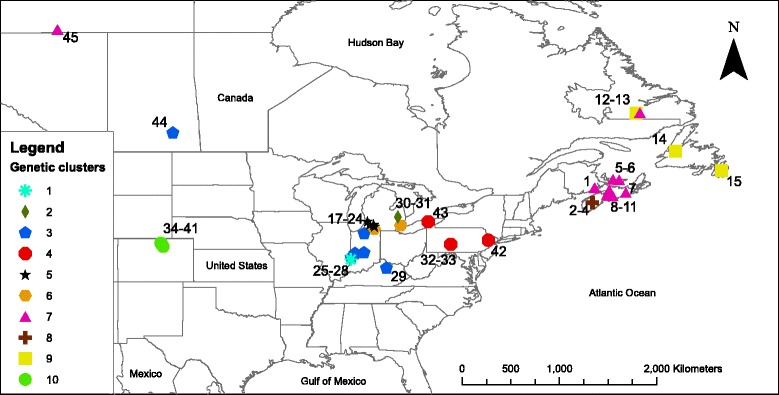
Sampling locations of *Cimex adjunctus* in North America. Created with ArcGIS v10.3 (ESRI, Redlands, USA). Numbers on the map correspond to site numbers in Additional file 1: Table S1. Membership to each of ten genetic clusters, defined using microsatellite data in Geneland, is shown with a unique colour and shape. Cluster numbers are given in the Legend and correspond to those in Additional file 1: Table S1

We collected 160 *Cimex adjunctus* samples from throughout its range in North America (108 from *E. fuscus*, 36 from *M. lucifugus* and 16 from *M. septentrionalis*; Fig. 1; in Additional file 1: Table S1), from 45 sites (Additional file 2: Table S2). We successfully amplified a fragment of the cytochrome c oxidase 1 (*CO1*) gene for 154 *C. adjunctus*, and identified 41 haplotypes with 46 polymorphic sites (data available in Additional file 1: Table S1). We also genotyped 150 of the *C. adjunctus* at nine microsatellite loci (data available in Additional file 3: Table S3). We successfully obtained both mitochondrial and microsatellite data for 144 *C. adjunctus* (94% of the *CO1* dataset and 96% of the microsatellite dataset; in Additional file 1: Table S1). For approximate Bayesian computation (ABC), which uses both types of markers, we used the overlapping dataset (144 individuals). For analyses using only microsatellite loci (genetic clustering and isolation by distance, IBD), we used the whole microsatellite dataset (150 individuals) and for analyses using only mitochondrial DNA (minimum spanning network, MSN; mismatch distribution, MD; and extended Bayesian skyline plot, EBSP), we used the complete *CO1* dataset (154 individuals). Finally, we used only sites with data for at least two individuals, and for which we obtained both mitochondrial and microsatellite data, for the analysis of molecular variance (AMOVA) analysis, which resulted in a dataset of 127 individuals from 26 sites (82% of the *CO1* dataset and 85% of the microsatellite dataset).

### Microsatellite diversity, and Hardy-Weinberg and linkage disequilibrium

Among the nine microsatellite loci, we observed between two and 31 alleles. Across different sites and genetic clusters (identified by Geneland), average number of alleles ranged from 1.5 to 4, expected heterozygosity ranged from 0.18 to 0.62, observed heterozygosity ranged from 0.09 to 0.25, and the inbreeding coefficient varied between 0.00 and 0.77 (Table 1). Variation in genetic diversity and inbreeding coefficients did not show any obvious spatial pattern. We found three significant cases of deviation from Hardy-Weinberg equilibrium (one site at the loci Clec104 and Cle015, and another site at Clec104). Since these incidences of deviation from Hardy-Weinberg equilibrium were not systematic across loci or sites, we retained these two markers and two sites for our analyses. We did not find any evidence of significant linkage disequilibrium in any marker.

**Table 1:** Genetic diversity estimates for *C. adjunctus*, averaged across nine microsatellite markers, for sites with five or more sampled individuals and for genetic clusters identified by Geneland (with the exception of Cluster 1, in which there was only one individual; in Additional file 1: Table S1). Site and cluster numbers correspond to those in Fig. 1 and Additional file 1: Table S1

Site/Cluster	Average number of alleles	Expected heterozygosity	Observed heterozygosity	Inbreeding coefficient G_IS_
Site 17	2.000	0.275	0.278	−0.009
Site 19	1.889	0.363	0.093	0.745
Site 30	2.778	0.346	0.201	0.420
Site 31	2.111	0.327	0.254	0.223
Site 32	3.000	0.193	0.193	0.369
Site 36	2.444	0.325	0.224	0.310
Site 39	2.556	0.293	0.241	0.178
Site 40	2.222	0.239	0.145	0.393
Site 41	1.778	0.184	0.160	0.129
Cluster 2	3.000	0.349	0.225	0.431
Cluster 3	2.556	0.394	0.246	0.375
Cluster 4	3.222	0.332	0.235	0.291
Cluster 5	2.778	0.309	0.186	0.399
Cluster 6	2.222	0.327	0.247	0.245
Cluster 7	3.889	0.349	0.225	0.354
Cluster 8	3.000	0.615	0.143	0.768
Cluster 9	1.667	0.250	0.194	0.222
Cluster 10	3.556	0.301	0.196	0.349

### Range-wide genetic structure

Genetic clustering analyses using the Geneland method revealed 10 genetic clusters (Table 2), which were generally concordant with geographic location (Fig. 1). One interesting exception was that individuals from the Northwest Territories and Saskatchewan clustered with individuals from distant regions (Clusters 3 and 7; Fig. 1). There was no association between genetic clusters identified by Geneland and any major geographic barriers that might knowingly impact dispersal. The sampling year and host species did not seem to strongly affect clustering, as individuals associated with different host species or sampled at different years were frequently assigned to the same cluster (Additional file 4: Table S4). Using *K*-means clustering, we obtained the lowest BIC value at *K* = 11, and the second lowest BIC value was at *K* = 10. Moreover, we observed significant IBD calculated on individual genetic relatedness values (*P* = 0.001, R^2^ = 0.19; Table 2). Conditioning for genetic structure slightly improved the fit of the IBD model (R^2^ = 0.21; Table 2).

**Table 2:** Results of clustering and isolation-by-distance analyses of *Cimex adjunctus*, estimated using microsatellite markers. Most likely number of genetic clusters (K) estimated using the Geneland method, Isolation-by-distance (IBD) and IBD while correcting for population genetic structure (IBD + K) are shown

Statistic		Value
Most likely K		10
IBD (r_W_)	*P*	0.001*
	R^2^	0.19
IBD (r_W_) + K	*P* (IBD)	0.001*
	*P* (K)	0.001*
	R^2^	0.21

*Statistically significant at α = 0.05

AMOVA results were very different between the two types of markers. For microsatellites, considerably less of the total variation was explained by among (22.8%; Table 3) than within sites (37.0%), but for mitochondrial data the variation among (48.8%) and within sites (43.7%) were similar. The proportion of genetic variation among host species was high for microsatellite data (40.2%; Table 3), but quite low for mitochondrial data (7.4%).

**Table 3:** Results of analysis of molecular variance (AMOVA) on *Cimex adjunctus*, using mitochondrial and microsatellite data*.* Percentage of total variation among host species, among sample sites (population), and within sample sites are shown

Source of variation	Mitochondrial	Microsatelite
Among host species	7.4	40.2
Among populations	48.8	22.8
Within populations	43.7	37.0

### Demographic history

**Fig. 2:**
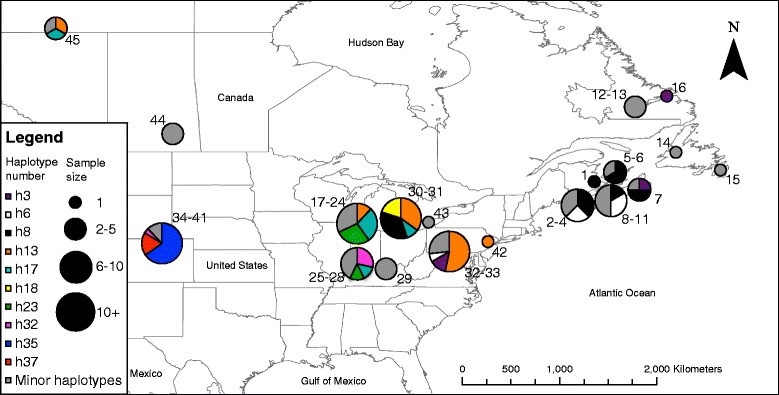
Frequencies of mitochondrial DNA haplotypes of *Cimex adjunctus* across its range. Data for nearby sampling sites are combined in a single pie chart. Rare haplotypes represented by fewer than four individuals in the entire data set are shown in grey. Haplotypes represented by four or more individuals in the entire dataset are identified with unique colors as indicated in the Legend, and corresponding to haplotype colors in Fig. 3. Site numbers correspond to those in Fig. 1 and in Additional file 1: Table S1. Sizes of circles indicate sample sizes

**Fig. 3:**
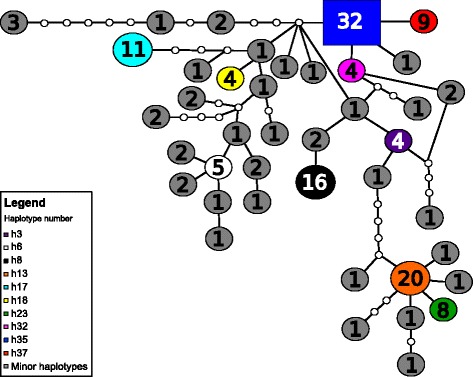
Minimum-spanning network of mitochondrial cytochrome c oxidase 1 (*CO1*) sequences of *Cimex adjunctus*. Haplotypes represented by fewer than four individuals are shown in grey. Haplotypes represented by four or more individuals are identified with unique colors as indicated in the Legend, and corresponding to those in Fig. 2. Each circle represents a unique sequence, each line segment is a mutational step, numbers are sample sizes for each unique sequence, small circles without a sample size are intermediate, unsampled haplotypes, and the square represents the putative ancestral sequence

**Fig. 4:**
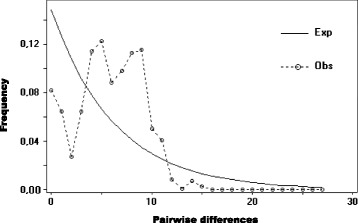
Frequency of pairwise mismatches among cytochrome c oxidase 1 (*CO1*) sequences of *Cimex adjunctus* in North America

**Fig. 5:**
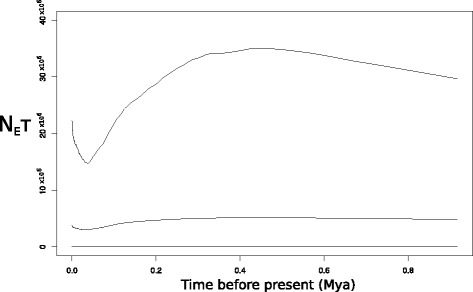
Extended Bayesian skyline plot estimated using cytochrome c oxidase 1 (*CO1*) data of *Cimex adjunctus*. Shown are the mean and 95% highest posterior density interval of the product of effective population size (N_E_) and generation time (τ) through time (in million years ago; Mya)

Considering those haplotypes represented by four or more individuals, there was some degree of spatial structuring in their distribution. Specifically, distinct haplotypes were associated with the western and eastern ends of *C. adjunctus’* range (Fig. 2). One interesting observation was that individuals from Northwest Territories and Saskatchewan had very similar haplotypes to individuals from the Midwest of the United States. The MSN did not show a well-defined starburst pattern (Fig. 3). Also, the MD showed multiple peaks rather than a single peak that would have indicated potential demographic expansion in the past (Fig. 4). Evolution of N_E_τ through time, estimated using EBSP, showed mostly constant population size with a possible gradual decrease from about 200,000 to 30,000 years ago to about half of the initial population size, followed by a small increase to the present (Fig. 5). Finally, ABC analysis gave strongest support to a scenario mimicking a decrease in effective population size of at least an order of magnitude between 10 million years ago and 10,000 years ago (Table 4; See Additional file 5: Figure S1 for pre-evaluation of prior distributions of scenarios with the observed values).

**Table 4:** Results of approximate Bayesian computation analysis of effective population size (N_E_) history of *Cimex adjunctus*. Posterior probabilities of each scenario (with confidence interval in parentheses) are shown

Scenario	Posterior probability
N_E_ Increase	0.297 (0.285 – 0.308)
N_E_ Decrease	0.522 (0.508 – 0.536)
N_E_ Constant	0.181 (0.167 – 0.196)

## Discussion

### Range-wide genetic structure

Analyses of mitochondrial and microsatellite genetic markers supported our prediction of high range-wide genetic structure, mediated by geographic distance, in *C. adjunctus*, an ectoparasite of bats. Across the range of *C. adjunctus*, we found significant genetic structure, a large proportion of which was explained by geographic distance. Whereas IBD has not been previously investigated in most bat parasites (but see [33]), it has been investigated in two of the key hosts of *C. adjunctus*, the big brown bat and the little brown myotis. A relationship between genetic and geographic distance has been observed in both the big brown bat [20] and little brown myotis across a considerably smaller spatial scale [24] than examined here. Range-wide IBD has also been described for little brown myotis [25], based on population-level analyses using *F*_ST_. Thus, geographic distance explains a lot of the variation in genetic structure of *C. adjunctus* as it does in two of its hosts, which could potentially reflect the reliance of *C. adjunctus* on their hosts for dispersal.

However, the overall degree of genetic structuring appears to be higher in *C. adjunctus* than in its hosts. Analysis of microsatellite genotypes has revealed only two genetic clusters in both big brown bat [34] and little brown myotis [25], both at continental spatial scales, whereas our results point to ten genetic clusters in *C. adjunctus*. Likewise, very little genetic variation (<10% with microsatellite data, and < 20% with mitochondrial data) occurs among spatially separate sites in big brown bat [20] and in little brown myotis [22–25]. In *C. adjunctus*, about one third of the microsatellite variation and about one half of mitochondrial variation occur among sites (after taking out variation among host species). These observations suggest that *C. adjunctus* is more subdivided within its range than at least two of its hosts, and that its genetic structure does not entirely reflect the dispersal patterns of its hosts. Interestingly however, both genetic clustering and MSN results also offer some evidence of possible continent-scale long-distance movement in *C. adjunctus*, as reflected in the relationships among individuals from the Northwest Territories, Saskatchewan, Maritime Canada and the US Midwest. Relationships among *C. adjunctus* samples from these locations echo a pattern that was observed in *M. lucifugus*, where a set of sites in the central United States and central to north-western Canada are connected by high gene flow [25].

Spatial structuring of genetic diversity can arise when gene flow is not sufficiently high to homogenize allele frequencies throughout the study area, and across a broad range of animal species dispersal ability is correlated with both gene flow and population genetic structure [3]. This has led to the prediction that genetic structure of many parasites will reflect host dispersal and genetic structure [10]. However, the association between host dispersal and parasite genetic structure has recently been shown to be generally weak [10]. Furthermore, genetic structure in parasites is often found to be stronger than that of their host, as we have observed here for *C. adjunctus*. For example, a finer genetic structure was found in an endoparasitic nematode H. *polygyrus* than in its host, the field mouse *A. sylvaticus* [8]. One reason for stronger genetic structuring in parasites than their hosts could be that, for parasites using their host as a means of dispersal, not every host dispersal event will result in dispersal by the parasite. This is likely to be the case for *C. adjunctus*, which spends a considerable proportion of time living off of its hosts within cracks and crevices in roosting sites. First, only a small subset of dispersing bats are likely to be accompanied by *C. adjunctus.* Second, dispersal mortality in the parasite may be very high due to grooming behaviour of bats that can cause the parasites to fall off [35]. Additionally, parasites that have a generation time that is much shorter than that of their hosts, that are associated with more than one host species, or that are associated with highly mobile hosts typically show a much stronger genetic structure than their host, as highlighted by Mazé-Guilmo et al. [10]. All of these factors are true for *C. adjunctus*, and could explain the much stronger genetic structure we observed for relative to two of its key hosts.

In addition to gene flow and dispersal, genetic structure may also be influenced by genetic drift in small populations, which acts by increasing differentiation [36]. Bat-associated *Cimex* populations might be much smaller than populations of their hosts, although information on *C. adjunctus* population sizes is limited. In addition, it is possible that *C. adjunctus* experiences localized extirpations and recolonizations when roosts are abandoned by bats and subsequently re-occupied. The resulting founder events would further reduce effective population sizes and lead to higher genetic differentiation in *C. adjunctus* via genetic drift.

We also examined the proportion of genetic variance among samples of *C. adjunctus* associated with different host species. Interestingly, we found a sharp difference between mitochondrial DNA and microsatellite markers in this regard. Mitochondrial data suggested considerably less variation among populations associated with different host species compared to microsatellite data. At the same time, microsatellite data showed less variation among populations than did the mitochondrial data, indicating that the difference we observed with respect to host species does not reflect a generally poorer ability of the mitochondrial data to detect differentiation in *C. adjunctus*.

Our mitochondrial data are consistent with an earlier study on *C. pipistrelli* that found no genetic differentiation among individuals associated with different host species, using mitochondrial *CO1* and four nuclear loci [30]. Our microsatellite results contradict these results from *C. pipistrelli*, although it is important to point that all nuclear loci in the study of Balvin et al. [30] showed almost no variation. Mitochondrial DNA is maternally inherited and therefore variation in it will reflect dispersal and history of the maternal lineage only. It is possible therefore that sex-biased behaviour in *C. adjunctus* could be the reason for our results. Male-biased dispersal among roosts could lead to the higher proportion of genetic variation among sites in mitochondrial data than in microsatellite data. On the other hand, female-biased switching of hosts within roosts could be responsible for the lower proportion of genetic variation among host species observed in the mitochondrial versus microsatellite data. Autonomous (i.e., not host-assisted) female-biased movements over short distances, such as between neighbouring apartment units, have been described in the common bed bug, *C. lectularius* [37]. If female *C. adjunctus* also move more readily at short distances within roosts, that could explain both a higher rate of host-switching among females and a lower rate of transport among roosts by their hosts (since females might spend more time off of the hosts while they engage in exploratory behaviour). However, there is currently no information available on sex-biased dispersal or host switching in *C. adjunctus*. Our results not only suggest sex-biased dispersal or host switching in *C. adjunctus*, but also highlight the need to use more than one type of marker when investigating genetic diversity in an understudied species.

The most well studied member of the genus *Cimex* is the human associated common bed bug, *C. lectularius*. Several studies have examined genetic structure in *C. lectularius* across a range of spatial scales [38–42]. However, most such studies focus on a considerably smaller scale than we do here, making direct comparisons of genetic structure difficult. For example, Saenz et al. [42] describe a weaker IBD pattern in *C. lectularius* than we observed for *C. adjunctus*, which could be due in part to the smaller spatial scale of their sampling (eastern USA only). On the other hand, our genetic diversity estimates for *C. adjunctus* were strikingly similar to those found in one study on *C. lectularius* [41], although we report slightly higher average numbers of alleles. In an interesting parallel, a study of *C. lectularius* populations associated with bats and humans found higher average numbers of alleles in the bat-associated populations than human-associated populations [38]. Another study of *C. lectularius* in Europe [39] found higher mitochondrial DNA variation among bat and human associated populations than we observed among populations of *C. adjunctus* associated with different bat species. One likely reason for this dissimilarity between *C. adjunctus* and *C. lectularius* is that the former is a weak generalist, associated with closely related species [10], while the former is a strong generalist, associated with phylogenetically very different species. Overall, sample sizes and the number of microsatellite markers used were lower in our study than in several studies of *C. lectularius* genetic structure [34, 40, 41], but were nonetheless appropriate given the much broader spatial and temporal scale of resolution of our analyses [5, 8, 12, 43].

### Demographic history

We predicted signals of range and demographic expansion in *C. adjunctus*, based on the fact that there are widespread signatures of historic population expansion in many vertebrates, invertebrates and plant populations, including in the bat hosts of *C. adjunctus*. Such patterns are most probably attributable to postglacial climate warming [44]. However, we found that the history of this ectoparasite is marked most strongly by demographic decline, with only a weak signal of recent demographic expansion, and no clear pattern of range expansion. For example, typical starburst patterns were previously observed in the haplotypic networks of *E. fuscus* and *M. lucifugus* [21–23], indicative of range expansion. However, we found no clear starburst pattern for *C. adjunctus*. This is unlikely to be a result of inadequate spatial sampling since our samples cover most of the known range of this species [14].

We found evidence of population decline in the demographic history of *C. adjunctus* using a variety of approaches. According to EBSP results, a gradual decline might have started at around 200,000 years ago, corresponding roughly to the Illinoian glaciation, a time of likely very harsh climate for most species in North America [45]. A small demographic recovery may have started at around 30,000 years ago. Our ABC results confirmed a population decline as the most likely historical scenario. Two previous studies found signals of demographic expansion in *M. lucifugus* in eastern Canada [22] and Minnesota, United States [46]. A small potential increase in *C. adjunctus* effective population size indicated in the EBSP starting 30,000 years ago is in a similar timeframe as, but is of much smaller amplitude than, the demographic expansion found in both *M. lucifugus* studies. Relative to those studies, our analysis was able to span a larger amount of time, probably due to the larger spatial scale of our sampling.

## Conclusions

Parasites that are mostly free-living, associate with multiple species of hosts, and have hosts that are highly mobile, such as some ectoparasites of bats, may be expected to show a genetic structure that contrasts with the dispersal patterns and genetic structure of their hosts [10]. These same factors may also lead to a difference in historic patterns of change in host and parasite ranges and population sizes. We have found exactly this pattern in *C. adjunctus*, an insect ectoparasite associated with a number of bat species in North America. This free-living parasite moves off the host between blood meals and could be actively removed by the host through anti-parasitism behaviour. Our results highlight that the genetic structure and demographic history of a weak generalist ectoparasite, particularly one that has a loose relationship with its hosts, can be very different from that of its hosts.

## Methods

### Sample collection

We collected *C. adjunctus* across much of its North American range. Most samples are from mist-netted host individuals of *E. fuscus*, *M. lucifugus* or *M. septentrionalis*. Mist net capture locations were adjacent to a known summer roost (house, barn, cabin, church, school or abandoned mine) of either of the three bat species, or within forested national, provincial, state or territorial lands (Additional file 2: Table S2). Most mist-netted bats and the *C. adjunctus* individuals they harboured likely came from the adjacent known roost, although it is possible that a small proportion came from different roosts in the area. Overall, between 3 and 15% of mist-netted bats harboured a parasite, depending on the location*.* We also sampled *C. adjunctus* individuals from the interior of two summer roosts. One roost was in a church attic inhabited by *M. lucifugus*, and one was in a house attic inhabited by *E. fuscus* (Additional file 2: Table S2). Because we could be certain of the roost site in these cases, we considered these two sampling locations as distinct from their adjacent mist-netting capture locations. Upon collection, we stored samples immediately in a 95% ethanol solution until further analyses. We then generated *CO1* mitochondrial DNA sequence data and nine nuclear microsatellite genotype data for all individuals. All samples included in this study were confirmed as being *C. adjunctus* using a DNA barcoding approach [47]. We compared the *CO1* sequence for each sample to known *CO1* sequences for *Cimex* species from published sources [48].

### Genetic analyses

We extracted DNA from the whole insect for all samples using the DNeasy Blood & Tissue Kit (QIAGEN, Germantown, Maryland, United States). We then amplified a 576-bp fragment of the *CO1* gene from each individual using the primers: F 5’- TATGAGCAGGCATGTTAGGG and R 5’-ATAGATGTTGATAAAGAATTGGG (Designed by our group based on published sequences of Balvin et al. [48]). We used a DNAEngine PTC-200 Thermal Cycler (BIO-RAD, Hercules, California, United States) to execute the Polymerase Chain Reaction (PCR) amplification. We performed PCR in 25 μL final volume using the following recipe: 1X Taq Polymerase Buffer excluding MgCl_2_ (Applied Biosystems, Foster City, California, United States), 1.5 mM of MgCl_2_, 0.2 mM of each type of dNTP, 0.3 μM of each primer, 1 U of Taq polymerase (ABI), and 1 μL of DNA extraction product. We used the following PCR program: an initial denaturation step of 1 min at 94 °C, followed by 36 cycles of 30 s of denaturation at 94 °C, 45 s of annealing at 49 °C and 45 s of extension at 72 °C, finished by a final extension step of 5 min at 72 °C. We visualized PCR products by 1.5% agarose gel electrophoresis using SYBR Green (BIO-RAD) on a UV transluminator to check the quality and size of amplified fragments. Then, we sequenced the amplified gene fragment for every sample using Sanger sequencing with BigDye terminator chemistry (ABI) and analyzed the fragments on a 3730xl DNA Analyzer (ABI). We aligned all sequences using MEGA 6.06.

We also genotyped all individuals at nine microsatellite loci originally designed for *Cimex lectularius* (Cle002, Cle003, Cle013, Cle015, from Fountain et al. [40], and Clec21, Clec48, Clec15, Clec104 and BB28B, from Booth et al. [42]; in Additional file 6: Table S5). We used a DNAEngine PTC-200 Thermal Cycler (BIO-RAD) to execute PCR amplification. For markers from Fountain et al. [41], we performed PCR using the following recipe: 1X Taq Polymerase Buffer excluding MgCl_2_ (ABI), 2.175 mM of MgCl_2_, 0.216 mM of each type of dNTP, 0.25 to 1.2 μM (Additional file 6: Table S5) of each primer, 1 U of Taq polymerase (ABI), 2 μL of DNA extraction product, in total volume of 12 μL. For markers from Fountain et al. [40], we used the following thermal cycling: an initial denaturation step of 15 min at 95 °C, followed by 11 cycles of 30 s of denaturation at 94 °C, 1 min and 30 s of annealing (initially at 65 °C and reduced 1 °C at every cycle) and 1 min of extension at 72 °C, followed by 26 cycles of 30 s of denaturation at 94 °C, 1 min and 30 s of annealing at 55 °C and 1 min of extension at 72 °C, finished by a final extension step of 10 min at 72 °C. For markers from Booth et al. [41], we used the following thermal cycling: an initial denaturation step of 3 min at 95 °C, followed by 35 cycles of 30 s of denaturation at 95 °C, 30 s of annealing at 59 to 61 °C (Additional file 6: Table S5) and 30 s of extension at 72 °C, and a final extension step of 5 min at 72 °C. We amplified each locus individually. PCR products were visualized by 1.5% agarose gel electrophoresis using SYBR Green (BIO-RAD) on a UV transluminator to check the quality and size of amplified fragments. We then sized products on a 3730xl DNA Analyzer (ABI). We called all microsatellite genotypes for each species using GeneMapper Software 4.0 (ABI), and we checked all calls manually.

### Statistical analyses

#### Microsatellite diversity, and Hardy-Weinberg and linkage disequilibrium

For sites with data for at least five sampled *C. adjunctus* individuals, and for genetic clusters (see next section), we calculated average number of alleles, expected and observed heterozygosity, and inbreeding coefficient. For microsatellite loci, we tested for Hardy-Weinberg and linkage disequilibrium within each site with data for at least two sampled *C. adjunctus* individuals, using Genepop 4.2. For each type of test, we corrected for multiple tests using Bonferroni correction, with a threshold α of 0.05.

#### Range-wide genetic structure

We tested our prediction of range-wide genetic structure and an effect of geographic distance in *C. adjunctus* using genetic clustering, tests of isolation-by-distance (IBD), and an analysis of molecular variance (AMOVA). We conducted a Bayesian clustering analysis using Geneland 4.0.5, which takes into account geographic coordinates of individual samples. We used 100,000 iterations, thinned every 100^th^ iteration, and a post-process burn-in of 200 (of the 1000 left after thinning), for *K* values between 1 and 20. We executed 10 runs, and kept the one with the highest posterior mean density, after burn-in. We attempted to identify the population to which each individual was assigned the most often, defined here as the population where the majority of Markov Chain Monte Carlo (MCMC) chains converged for any given individual. We also conducted a *K*-Means clustering analysis using GenoDive 2.0 on allele frequencies, for *K* values between 1 and 20, and using 50,000 simulation steps, to validate results obtained with the Geneland method. We used Bayesian Information Criterion (BIC) values to determine the most likely *K* value.

We conducted an individual-level analysis of IBD, using the estimate of genetic relatedness, r_W_ [49], calculated with SpaGeDi 1.5. We calculated 1 – r_W_ for each pairwise relationship, in order to obtain genetic distances. We calculated geographic distance (in km) between sample sites, corrected for sphericity of the earth, using the ‘rdist.earth’ function from the ‘fields’ package [50] in R v3.1.3 (R Development Core Team, Vienna, Austria). We then fit pairwise genetic distance to geographic distance using Multiple Regression on distance Matrices (MRM), in the ‘MRM’ function from the ‘ecodist’ package in R v3.1.3 [51], which uses a Mantel test derived linear regression model. We assessed significance through a permutation procedure (9999 replicates). An assumption of the r_W_ relatedness index, and most other relatedness indices, is that individuals are in a large random mating population without population structure [52]. In an attempt to correct for the population structure present in our dataset, we subsequently conditioned IBD models for genetic clustering. For each pair of individuals assigned to the same population in clustering analyses, we assigned a value of 0, and for each pair of individuals assigned to different populations, we assigned a value of 1. We then tested the effect of geographic distance, together with genetic clustering, on genetic distance in an MRM model.

For all sites with at least two sampled individuals, we used AMOVA to examine the proportion of genetic variation among sites, and among individuals associated with different host species. AMOVA was executed in GenoDive 2.0 for microsatellite data, and Arlequin 3.5 for mitochondrial data.

#### Demographic history

We tested the prediction that *C. adjunctus* would show signals of demographic and range expansion, similar to some of its bat hosts, with a suite of methods for investigating demographic history using either mitochondrial data alone, or both mitochondrial and microsatellite data. First, we produced a minimum-spanning network of mitochondrial haplotypes (MSN) using TCS 1.21, with a 95% connection limit. MSNs can indicate past range expansions if they show starburst like patterns [53, 54]. We expected to find such evidence pointing towards range expansion in *C. adjunctus*.

We executed a Mismatch Distribution (MD) analysis with DNASP 5.1. The purpose of this analysis is to compare the distribution of the frequency of each number of pairwise mitochondrial sequence mismatches in the dataset to the expected distributions under demographic expansion or constant population size through time. A unimodal peak at a non-zero number of pairwise mismatches is associated with demographic expansion, which we expected to observe, whereas more than one non-zero number of pairwise mismatches is usually associated with a constant population size through time [55].

Then, we constructed an Extended Bayesian Skyline Plot (EBSP) using mitochondrial data in BEAST 1.8.4. We used a linear EBSP model, and random local clock, which reportedly performs better than strict and relaxed clocks for most situations using intraspecific data [56, 57]. In trial runs, we found the HKY substitution model [58] to be the best-fitting model, as has also been shown for *Triatoma infestans* [59], a species in a genus closely related to *Cimex*. We used the gamma sites model to account for heterogeneity of substitution rate among individual loci. We used the default value of 10,000,000 Markov Chain Monte Carlo (MCMC) chains, logging every 1000 chains. We set the substitution rate to 0.575%/Ma, or half of 1.15%/Ma, which is the standard Arthropod mitochondrial pairwise substitution rate as reported by [60]. All other parameters were kept at default value. EBSPs allow one to visualize effective population size (N_E_) multiplied by generation time (τ) since some time in the past. In the case of highly structured populations, Heller et al. [61] suggested that a pooled sampling scheme, where several individuals are taken from about ten populations, was ideal to avoid a confounding effect of population structure, as opposed to all samples taken from the same population or one sample taken for each of a large number of populations. The sampling scheme used in our analysis fits well with the described pooled scheme. We expected to see an increase in effective population size over time, corresponding with a post-Pleistocene climate warming timeline.

Finally, we executed approximate Bayesian computations (ABC) on both mitochondrial and microsatellite data, using DIYABC 2.1.0. ABCs allow one to compare posterior probabilities of different demographic scenarios [62]. As per the method of [63], we input three scenarios in the analyses (for population sizes N_A_ > N_1_ > N_B_), mimicking an increase in effective population size from N_1_ to N_A_ at time *t*, a decrease in population size from N_1_ to N_B_ at time *t*, and finally constancy in population size at N_1_. Boundaries for N_A_, N_B_, N_1_ and *t* priors are available in Table 5. We set the potential time for the population size change event between 10,000 and 10 million years ago, to encompass a broad period of major climatic changes in the northern hemisphere [64]. We set the upper boundary of effective population after an increase (N_A_) to 10 times the initial upper boundary of effective population size (N_1_), to limit our analysis to population size increases of at least an order of magnitude. Similarly, we set the lower boundary of effective population size after a decrease (N_B_) to 1/10 of the initial lower boundary of effective population size (N_1_) to limit our analysis to population size decreases of at least an order of magnitude. If no change in population size of at least an order of magnitude occurred in the analysis timeframe, or if both a population size decrease and increase of similar magnitude occurred, then the scenario of constant population size would be most likely. We conducted a series of initial trial runs to determine the effective population size parameters for ABC analysis where we could achieve convergence between priors and observed values (Table 5). As an example, our final effective population size parameter values are large in comparison with those in a study on the invasive ladybird *Harmonia axyridis* [65]. Trial runs also indicated the best fit was achieved when we used a mutation rate per site between 10^−7^ and 10^−9^, with a mean at 10^−8^, for both mitochondrial and microsatellite markers, and the Stepwise Mutation Model (setting coefficient P at 0) for microsatellite markers. The estimated mutation rate for microsatellites is on the low end for such markers, but consistent with the relatively low variability observed for markers originally developed in a different species (i.e., ascertainment bias; [66]). We used “Mean number of alleles” and “Mean genic diversity” as summary statistics for microsatellite loci, and “Number of haplotypes”, “Mean of pairwise differences” and “Private segregating sites” for the mitochondrial locus. We computed 3,000,000 simulated datasets to compare with the observed dataset. First, we pre-evaluated the fit of observed values to prior distributions of scenarios, using a Principal Component Analysis implemented with the software. In a graph of the first two principal components, a good prior assessment is reflected in the observed values being approximately in the centre of the prior values for all three scenarios. Second, we calculated posterior probabilities for all three scenarios using a logarithmic regression, to determine which scenario is the most likely given the data. We expected strong support for a scenario mimicking a demographic expansion.

**Table 5:** Parameter values used in the approximate Bayesian computation analysis of demographic history of *Cimex adjunctus*. The set lower and upper boundaries of the three effective population size parameters are shown: N_1_ is the effective population size before population size change, N_A_ is the effective population size after demographic expansion, and N_B_ is the effective population size after demographic decline. The time period over which a population size change potentially occurred is *t* (in years)

Parameter	Lower boundary	Upper boundary
N_A_	500,000	50,000,000
N_B_	50,000	5,000,000
N_1_	500,000	5,000,000
*t*	10,000	10,000,000

## Additional files

Additional file 1: Table S1. List of *Cimex adjunctus* specimens included in analyses. Site refers to each unique sampling location in the study. Host species refers to the bat species from which the samples were collected, or which was inhabiting the roost from which the samples were collected (EPFU : *Eptesicus fuscus*, MYLU : *Myotis lucifugus*, MYSE : *Myotis septentrionalis*). Individuals with the same haplotype number share the same mitochondrial *CO1* haplotype sequence (we could not obtain *CO1* information for six individuals). We provide the Genbank accession number for each unique haplotype the first time it appears in the table. The genetic cluster (identified by Geneland based on microsatellite data) to which each specimen was assigned is also given (we could not obtain microsatellite data for 10 individuals). (XLSX 51 kb)
Additional file 2: Table S2. Details of sampling locations where we collected *Cimex adjunctus* specimens used in this study. Site refers to each unique sampling location and correspond to those in Additional file 1: Table S1. Sample size refers to the number of specimens collected at each site. Capture method refers to the way *C. adjunctus* samples were collected: either from the body of a bat that was captured outside a roost with a mist net or a harp trap (Bat capture), or from inside of the roost itself (Roost visit). Site characteristics refers either to the type of roost (house, barn, cabin, church or abandoned mine) in, or next to which, we collected samples, or to the forested land jurisdiction where collection was performed. (XLSX 37 kb)
Additional file 3: Table S3. Genotypic data at 9 microsatellite markers for 150 *Cimex adjunctus* individuals (we could not obtain microsatellite data for 10 individuals). The first three digits in each string describe the length (in base pairs) of the first allele, and the last three describe the length of the second allele. Missing data is identified with “000000”. (XLSX 59 kb)
Additional file 4: Table S4. Host species and sampling year from which *C. adjunctus* samples assigned to each genetic cluster were collected. (XLSX 41 kb)
Additional file 5: Figure S1. First and second principal components (percentage of explained variation in parentheses) of simulated values under each putative demographic scenario for *C. adjunctus*, as determined by approximate Bayesian computation (ABC), and comparison with observed values. (PDF 2969 kb)
Additional file 6: Table S5. Sequence, primer concentration ([Primer]), annealing temperature (T°), total number of alleles, and size range information for nine microsatellite markers previously designed for *Cimex lectularius* (by 1: Fountain et al. [41] and 2: Booth et al. [42]), used here on *Cimex adjunctus* samples. For each marker, we also show the average number of alleles (N_A_), expected heterozygosity (H_E_), observed heterozygosity (H_O_) and inbreeding coefficient (G_IS_) across all sites with five or more sampled individuals. (XLSX 51 kb)
