# Supplementary figures and images for: Range-wide genetic structure and demographic history in the bat ectoparasite Cimex adjunctus
Source: BMC Evol Biol. 2016 Dec 7;16:268. doi: 10.1186/s12862-016-0839-1 (PMC5142389; doi:10.1186/s12862-016-0839-1)

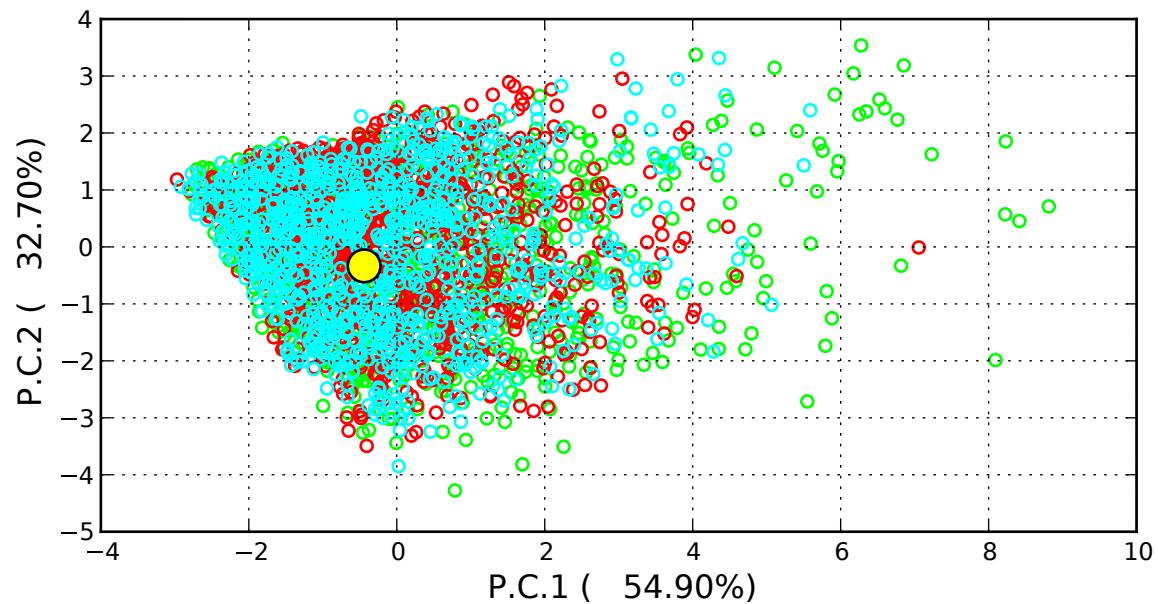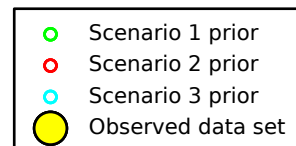

Supplement: Additional file 5: Figure S1. — First and second principal components (percentage of explained variation in parentheses) of simulated values under each putative demographic scenario for C. adjunctus, as determined by approximate Bayesian computation (ABC), and comparison with observed values. (PDF 2969 kb) [file 12862_2016_839_MOESM5_ESM.pdf]
